# Echoes of the Sarajevo Declaration on integrity and visibility of scholarly publications

**DOI:** 10.3325/cmj.2017.58.194

**Published:** 2017-04

**Authors:** Simeon Grazio, Branimir Anić, Frane Grubišić

**Affiliations:** 2 Editor-in-Chief of Liječnički vjesnik; School of Medicine, University of Zagreb, Zagreb, Croatia; 3 Editor-in-Chief of Fizikalna i rehabilitacijska medicina; University Hospital Center Sisters of Mercy, University Department of Rheumatology, Physical and Rehabilitation Medicine, Zagreb, Croatia

*To the Editor:* We read with great interest the Sarajevo Declaration on Integrity and Visibility of Scholarly Publications published as the editorial in the Croatian Medical Journal (1). We appreciate the efforts made by the authors, Journal Editors Group comprised of the selected lecturers at the First Mediterranean Seminar on Science Writing, Editing & Publishing (SWEP 2016). We agree with the statements of the Declaration, which highlights the main problems with editing scholarly journals in non-mainstream science countries and provides incentive for improving their standards, integrity, and visibility. The point we wish to bring forth is that the Declaration omitted to refer to journals published in languages other than English. We understand that these journals are less visible and out of focus of general scientific community, but given the main scope of the Declaration, we feel that it would have been valuable mentioning that the Declaration applies not only to journals published in English but also to journals published in other languages. The reason is that the problem of poor management, lack of understanding of how indexing services operate, and corruptive and predatory practice are even more manifest in these journals, which are vastly not covered by any kind of surveillance, such as Jeffrey Beall’s List of Predatory Publishers (2). Therefore, our efforts should be directed at all scientific journals, including those published in national languages. We believe that this should also be clearly stated in the Declaration.

We thank the authors for the Declaration and feel that, with our suggestion taken into account, we can enforce the ultimate goal of the Declaration, which is to improve scientific and publishing practice.
